# Why are hedonists less happy than eudaimonists? The chain mediating role of goal conflict and mixed emotions

**DOI:** 10.3389/fpsyg.2023.1074026

**Published:** 2023-02-20

**Authors:** Wujun Sun, Lei Liu, Yuan Jiang, Ping Fang, Xiaosheng Ding, Guangjun Wang

**Affiliations:** ^1^Faculty of Education, Henan Normal University, Xinxiang, Henan, China; ^2^School of Labor and Human Resources, Renmin University of China, Beijing, China; ^3^Department of Psychology, Beijing Sport University, Beijing, China; ^4^Department of Psychology, Capital Normal University, Beijing, China; ^5^Department of Physical Education, Beijing Union University, Beijing, China

**Keywords:** hedonic motivation, eudaimonic motivation, goal conflict, mixed emotions, life satisfaction

## Abstract

**Introduction:**

Human motivation for happiness involves two main orientations of hedonia and eudaimonia. Numerous studies have shown that hedonic motivation has a much smaller effect on happiness outcomes than eudaimonic motivation, but little is known about the reasons for this. According to the Self-Determination Theory and the Levels of Valence Model, this may be related to the different goal conflicts and mixed emotions elicited by the two motivations. To demonstrate this, the study examined the mediating effect of the above two variables between happiness motivation and life satisfaction. Furthermore, it explained why hedonists are less happy than eudaimonists by comparing the two happiness motivations in terms of their respective path effects.

**Methods:**

The study randomly selected 788 college students from 13 different provinces of China to examine the relationships between hedonic motivation, eudaimonic motivation, goal conflict, mixed emotions, and life satisfaction.

**Results:**

The result showed that (1) the direct effect of hedonic motivation on life satisfaction was marginally significant, and the effect size was much smaller than that of eudaimonic motivation. (2) The direct and indirect effects of hedonic motivation were the opposite, with a large suppressing effect. In contrast, all paths of eudaimonic motivation positively affected life satisfaction. (3) Hedonic motivation negatively influenced life satisfaction through mixed emotions and the chain mediating effect of goal conflict and mixed emotions, whereas eudaimonic motivation positively influenced life satisfaction through these two mediating paths. (4) The effects on all paths of hedonic motivation were significantly smaller than those of eudaimonic motivation, except for the path mediated by goal conflict.

**Discussion:**

This study explains why hedonists are less happy than eudaimonists from the perspective of goal pursuit, emphasizes the critical role of differences in goal pursuit state and experience between happiness motivation and life satisfaction, and provides new ideas for the study of the influence mechanism of happiness motivation. At the same time, the deficiencies of hedonic motivation and the advantages of eudaimonic motivation presented by the study provide directions for cultivating happiness motivation for adolescents in the practice field.

## 1. Introduction

What kind of lifestyle can make people happier, enjoyment or striving? Human exploration of this question has a long history, resulting in two different perspectives, hedonism, and eudaimonism (Gentzler et al., 2021; Sun et al., 2022). Hedonism asserts that happiness comes from the pursuit of pleasure and the avoidance of pain and that people can achieve the experience of happiness through timely enjoyment. In contrast, eudaimonism emphasizes self-actualization, the acquisition of meaning, and the fulfillment of potential as essential elements of happiness. Therefore, people have to struggle to achieve happiness through self-effort. Huta et al. argue that the two different lifestyles reflect the paths individuals take in their daily lives to pursue happiness based on different motivations (Huta and Ryan, 2010; Huta and Waterman, 2014). Focusing on the happiness motivations behind lifestyle will help to explore better the mechanism underlying people’s pursuit of happiness, including the power source, pathways, and outcomes. Happiness motivations reflect the intentions or purposes of individual daily activities, including both hedonic and eudaimonic motivations (Huta and Ryan, 2010). Numerous studies have shown that the relationship between hedonic motivation and happiness outcome is unstable compared to eudaimonic motivations, but little is known about the causes (Ortner et al., 2018; Lin and Chan, 2020; Sun et al., 2022). This study attempts to identify why hedonists are less happy than eudaimonists from the perspective of goal pursuit by comparing the differences in goal pursuit states and experiences dominated by the two motivations and exploring their differences in influencing the experience of happiness.

### 1.1. Hedonic motivation, eudaimonic motivation and life satisfaction

As an essential component of happiness and a key parameter for measuring life quality, life satisfaction has been widely used in studies of the relationship between happiness motivation and outcomes (Sun et al., 2021). Empirical studies have found that although both hedonic and eudaimonic motivation plays a vital role in predicting life satisfaction, the effect of the former is much less stable and long-lasting than that of the latter. Specifically, hedonic motivation is much less positively related to life satisfaction than eudaimonic motivation (Ortner et al., 2018; Braaten et al., 2019). Furthermore, hedonic motivation does not always effectively predict individual satisfaction, whereas eudaimonic motivation does (Tandler et al., 2020). The correlations and predictive relationships between hedonic motivation and life satisfaction are weaker in eastern cultural groups represented by China (Lin and Chan, 2020; Sun et al., 2022) than in western cultural groups (Ortner et al., 2018; Gentzler et al., 2021). These empirical results suggest that the effect of hedonic motivation on life satisfaction is not robust, especially in the Chinese cultural context. Accordingly, this study proposes hypothesis 1: Hedonic motivation has a much smaller effect on life satisfaction than eudaimonic motivation. This hypothesis is an expected replication of the prior findings.

Theoretical research on happiness motivation has argued that hedonic and eudaimonic motivations play a crucial role in predicting happiness outcomes because they represent the underlying reasons for individuals’ life goal choices and actions (Huta and Waterman, 2014; Giuntoli et al., 2021). Self-Determination Theory also suggests that factors related to goal pursuit play an essential role in the relationship between motivation and individual quality of life. For example, better health, overall happiness, and high energy levels are strongly related to whether an individual’s goal needs are met (Ryan and Deci, 2017). The difference in the effects of hedonic and eudaimonic motivation may be related to their different effects on life goal pursuit state and experience. Explaining this difference from the perspective of goal pursuit may provide us with new ideas to gain insight into the issue of the influencing mechanism of happiness motivation.

### 1.2. The role of goal conflict in happiness motivation and life satisfaction

According to Self-Determination Theory, the disadvantage of hedonic motivation compared with eudaimonic motivation may be that the process of its dominant goal attainment is not smooth, and goal needs are challenging to satisfy fully (Ryan and Deci, 2017; Giuntoli et al., 2021). Hedonic motivation takes pleasure and comfort as life goals, and there is often a tension between these goals and the effort required to achieve cognitive and academic goals in individual development. For example, Kryza-Lacombe et al. (2019) found that their eudaimonic motivation, rather than hedonic motivation, significantly predicted higher academic achievement among college students. Moreover, contrary to the beneficial effects of eudaimonic motivation on organizational and social goals, pursuing hedonic pleasure undermines realizing organizational and social goals. It thus is often not the desired behavior of organizations and societies, especially in eastern cultures that emphasize social obligations and collective interests (Yang et al., 2017; Lin and Chan, 2020). Therefore, individuals with high hedonic motivation are more likely to experience conflicts between hedonic and personal development goals, and between personal goals and organizational and social goals in their daily lives. The most recent motivation and personality theory suggests that when conflicting goals arise, the individual’s behavioral inhibition system is activated to evaluate risks and threats and inhibit goal behaviors (Corr and Krupić, 2017). At this time, individuals’ negative evaluations of the situation will increase, resulting in lower life satisfaction. Empirical evidence also shows a significant negative relationship between goal conflict and life satisfaction (Boudreaux and Ozer, 2013; Gray et al., 2017). Therefore, the two motivations have different potential effects on individuals experiencing goal conflict, and the goal conflict has a negative effect on life satisfaction. Thus, hypothesis 2 is proposed: Hedonic motivation may negatively influence life satisfaction mediated by goal conflict (Hypothesis 2a); whereas eudaimonic motivation may positively influence life satisfaction mediated by goal conflict (Hypothesis 2b).

### 1.3. The role of mixed emotions in happiness motivation and life satisfaction

Mixed emotions refer to the state in which both positive and negative emotions are experienced simultaneously and are more ecologically valid than pure positive or negative emotions alone in reflecting complex social contextual stimuli (Larsen, 2017). According to the Levels of Valence Model, stimulus situations that contain both positive and negative valence are necessary external conditions for generating mixed emotions (Shuman et al., 2013). The relaxation, excitement, and instant gratification sought by hedonic motivation, while leading to pleasurable experiences, are relatively short-lived and not always satisfying (Sheldon and Lyubomirsky, 2012). Therefore, hedonic motivation-oriented pursuit of happiness may contain positive and negative valence components and may encourage individuals to have mixed emotional experiences. In contrast, the engagement, achievement, and potential fulfillment sought by eudaimonic motivation contribute to the construction of enduring psychological resources that have a lasting positive effect on individual emotions (Waterman, 2007). Eudaimonic motivation-oriented pursuit of happiness often contain only positive valence, and rarely give rise to mixed emotions. Empirical results also show that hedonic motivation positively predicts positive and negative emotions, whereas eudaimonic motivation positively predicts positive emotions and negatively predicts negative emotions (Sun et al., 2021). The Co-activation Model of Healthy Coping suggests that in low to moderate stressful situations such as the pursuit of happiness, excessive mixed emotional experiences are detrimental to favorable health outcomes (Larsen et al., 2003). So mixed emotions tend to be accompanied by lower life satisfaction (Bee and Madrigal, 2013; Mejía and Hooker, 2017). Therefore, hypothesis 3 is proposed: Hedonic motivation may negatively influence life satisfaction mediated by mixed emotions (Hypothesis 3a); whereas eudaimonic motivation may positively influence life satisfaction mediated by mixed emotions (Hypothesis 3b).

### 1.4. The relationship between goal conflict and mixed emotions

The Levels of Valence Model suggests that conflict situations are essential for generating mixed emotions (Shuman et al., 2013). Boudreaux and Ozer (2013) showed that those with conflicting goals experienced more mixed emotions than individuals with facilitating goals. Laboratory and field experiments conducted by Berrios et al. (2015a) further demonstrated that goal conflict, whether artificially activated or naturally occurring, is a reliable predictor of mixed emotions. It is evident that goal conflict, as a typically mixed valence stimulus, is closely related to mixed emotions (Mejía and Hooker, 2017). Considering the close relationship between goal conflict and mixed emotions, the study hypothesized that happiness motivation might influence life satisfaction through the combined effect of goal conflict and mixed emotions. Therefore, this study proposed hypothesis 4: Hedonic motivation may negatively influence life satisfaction through the chain mediating effect of goal conflict and mixed emotions (Hypothesis 4a). In contrast, eudaimonic motivation may positively influence life satisfaction through this chain mediation effect (Hypothesis 4b).

**FIGURE 1:**
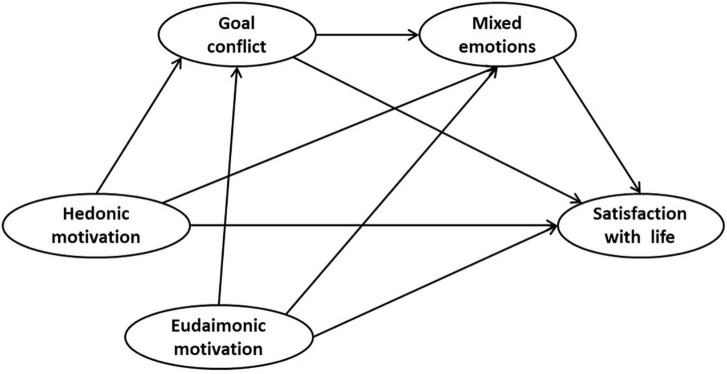
The theoretical model of chain mediation effect.

In conclusion, the relationship between hedonic motivation and life satisfaction is not robust compared to eudaimonic motivation, but the reasons for that are poorly understood. According to Self-Determination Theory, the effect of happiness motivation on life satisfaction may be related to its corresponding goal pursuit state and experience. Based on the contradictory nature of hedonic goals with individual development and social expectations, the study suggests that hedonic motivation is often accompanied by more goal conflicts and mixed emotions, which weakens its positive impact on life satisfaction. Therefore, goal conflict and mixed emotions may be the important mechanism and reason why hedonists are less happy than eudaimonists. According to this view, a chain mediating role model was constructed based on hypotheses 1–4 (Figure 1). The reasons for the significant effect variation between happiness motivations were explored by examine the differences in the nature and effect size of the different mediating paths of the two happiness motivations.

## 2. Materials and methods

### 2.1. Participants

The study recruited 788 participants from universities in 13 provinces across China, who completed an online questionnaire on Wenjuanxing platform.^1^ The age of the participants varied from 16 to 25 years, with a mean age of 19.93 (*SD* = 1.57). The sample included 436 males (55.3%) and 352 females (44.7%), with no significant differences in mean age (*M*_male_ = 20.02, *M*_female_ = 19.81, *t* = 1.92, *p* > 0.05). There are 229 freshmen (29.1%), 265 sophomores (33.6%), 184 juniors (23.3%), and 110 seniors (14.0%). 385 participants majored in social sciences (48.9%), and 403 participants majored in natural sciences (51.1%). Ethical approval for this study was obtained from the Human Subjects Ethics Branch of the University Research Committee of the corresponding author’s unit. All participants were informed of the voluntary and confidential nature of the study and provided their e-informed consent before completing the online questionnaire.

### 2.2. Measurements

#### 2.2.1. Hedonic and eudaimonic motives for activities questionnaire

The hedonic and eudaimonic motives for activities questionnaire compiled by Huta and Ryan (2010) was used to evaluate the extent to which people engage in activities based on hedonic and eudaimonic principles. The scale includes two subscales, hedonic motivation and eudaimonic motivation, with nine items. Five items (e.g., seeking relaxation) are used to measure hedonic motivation, and four items (e.g., seeking to pursue excellence or a personal ideal) are used to measure eudaimonic motivation. It is scored on a 7-point Likert scale, ranging from 1 (not at all) to 7 (very much), with higher scores on each subscale indicating a higher tendency toward hedonic or eudaimonic behavior. In this study, the fit indexes of the scale were *x*^2^/*df* = 4.818, RMSEA = 0.070, GFI = 0.971, NFI = 0.973, IFI = 0.978, TLI = 0.966, CFI = 0.978. The Cronbach’s α coefficient of the hedonic and eudaimonic motivation subscales were 0.864 and 0.893.

#### 2.2.2. Conflicting Goals Scale

The Conflicting Goals Scale developed by Berrios et al. (2018) was used to measure the degree of goal conflict experienced by individuals. The scale first asked participants to list their five most important current goals and then assessed the extent to which these goals have conflicted over the past few days using three items. For example, “I think that pursuing some of these goals hurts the pursuit of the other ones.” The scale uses a 5-level Likert scale, ranging from 1 “strongly disagree” to 5 “strongly agree,” with higher scores indicating higher levels of goal conflict experienced by the participants. The Conflicting Goals Scale is less time-consuming and more convenient than the Strivings Instrumentality Matrix, which has been commonly used to measure conflicting goals (Emmons and King, 1988; Sun et al., 2021). In this study, Cronbach’s α coefficient of the scale was 0.753.

#### 2.2.3. Mixed Emotions Scale

The Mixed Emotions Scale developed by Berrios et al. (2015a) was used to measure the participants’ emotional experiences during the past few days. The scale has four items, such as “I felt contrasting emotions.” A 5-point Likert scale ranged from 1 (not at all) to 5 (very strongly), with higher scores indicating higher levels of mixed emotions. This scale is a commonly used measure of mixed emotions and has been shown to have good reliability and validity (Berrios et al., 2015b). In the present study, the fit indexes of the mixed emotion scale were *x*^2^/*df* = 6.812, RMSEA = 0.086, GFI = 0.992, NFI = 0.993, IFI = 0.994, TLI = 0.983, CFI = 0.994, and the Cronbach’s α coefficient of the scale was 0.903, indicating good internal consistency.

#### 2.2.4. Satisfaction with Life Scale

Diener et al.’s (1985) Satisfaction with Life Scale was used to reflect the participants’ happiness evaluation. The scale consists of 5 items, e.g., “In most ways, my life is close to my ideal.” A 7-level Likert scale is used, ranging from 1 (strongly disagree) to 7 (strongly agree), with higher scores indicating higher levels of individual life satisfaction. In the present study, the fit indexes of life satisfaction scale were *x*^2^/*df* = 4.406, RMSEA = 0.066, GFI = 0.991, NFI = 0.993, IFI = 0.994, TLI = 0.986, CFI = 0.994, and the Cronbach’s α coefficient of the scale was 0.89.

### 2.3. Statistical analysis

Harman’s single factor test was used to test the common method biases for all items on the scales. SPSS 21.0 was used for descriptive statistics and correlation analysis. Amos 21.0 was used to test the chain mediation effect model. The percentile bootstrap method based on bias correction was chosen, and 5,000 bootstrap samples were sampled to obtain standard errors and bootstrap confidence intervals of parameter estimates.

## 3. Results

### 3.1. Common method biases test

Harman’s single factor test showed that seven factors with eigenvalues greater than one were extracted. Furthermore, the variance explained by the most significant common factor obtained before and after rotation was 28.64% and 18.48, respectively, which were less than the critical criterion of 40% (Podsakoff et al., 2003). Therefore, the influence of common method bias in this study is low.

### 3.2. Results of correlation analysis

In Table 1, the results of correlation analysis showed that hedonic motivation was positively correlated with eudaimonic motivation, goal conflict, mixed emotions, and life satisfaction. There was a significant negative correlation between eudaimonic motivation and mixed emotions, a significant positive correlation with life satisfaction, and a non-significant correlation with goal conflict. There was a significant positive correlation between goal conflict and mixed emotions and a non-significant correlation with life satisfaction. There was a significant negative correlation between mixed emotions and life satisfaction. Among the demographic variables, age was positively correlated with goal conflict, and gender was negatively correlated with life satisfaction.

**TABLE 1:** Descriptive statistics and correlation matrix of each variable (*n* = 788).

	*M*	*SD*	1	2	3	4	5	6	7	8	9
1. Age	19.93	1.57	1								
2. Gender	–	–	-0.07	1							
3. Grade	–	–	0.47***	-0.07	1						
4. Major	–	–	-0.10**	-0.12**	-0.08*	1					
5. Hedonic motivation	4.56	1.30	0.01	0.13**	0.01	-0.01	1				
6. Eudaimonic motivation	5.06	1.22	-0.05	0.05	-0.08*	-0.04	0.53***	1			
7. Goal conflict	2.98	0.86	0.08*	0.03	0.06	-0.03	0.13***	-0.03	1		
8. Mixed emotions	2.50	0.92	0.00	0.04	-0.05	-0.05	0.09*	-0.10**	0.39***	1	
9. Life satisfaction	4.16	1.35	-0.04	-0.10**	-0.05	-0.01	0.27***	0.39***	-0.07	-0.18**	1

*p < 0.05, **p < 0.01, ***p < 0.001.

### 3.3. Analysis of chain mediating model

**FIGURE 2:**
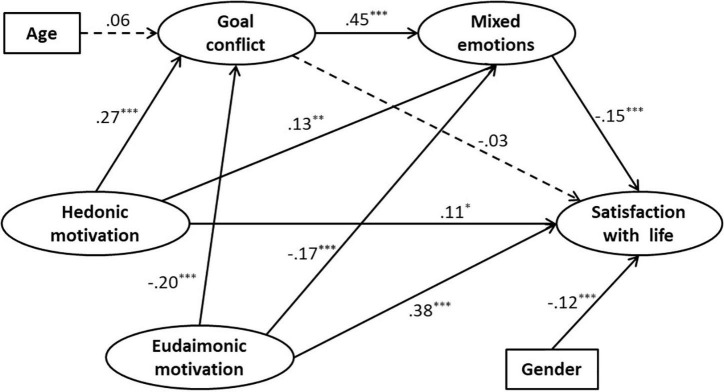
The chain mediating effect model of goal conflict and mixed emotions.

Based on the results of correlation analysis, Amos 21.0 was used for structural equation modeling analysis to test the mediation model proposed by the hypothesis. Since age is significantly correlated with goal conflict, and gender is significantly correlated with life satisfaction, the study included the two variables as control variables in the structural model. Data analysis results showed that the fit indexes of the mediation model were *x*^2^/*df* = 2.890, RMSEA = 0.049, GFI = 0.932, NFI = 0.935, IFI = 0.956, TLI = 0.950, CFI = 0.956, the theoretical model fit the data well. As shown in Figure 2, hedonic motivation significantly and positively influenced goal conflict (β = 0.27, *t* = 4.71, *p* < 0.001), mixed emotion (β = 0.13, *t* = 2.58, *p* = 0.01), and life satisfaction (β = 0.11, *t* = 2.22, *p* < 0.05). Eudaimonic motivation significantly and negatively influenced goal conflict (β = −0.20, *t* = −3.62, *p* < 0.001) and mixed emotions (β = −0.17, *t* = −3.59, *p* < 0.001), positively influenced life satisfaction (β = 0.38, *t* = 7.40, *p* < 0.001). Goal conflict significantly and positively influenced mixed emotions (β = 0.45, *t* = 9.25, *p* < 0.001), and its negative influence to life satisfaction was not significant (β = −0.03, *t* = −0.63, *p* > 0.05). Mixed emotions significantly and negatively influenced life satisfaction (β = −0.15, *t* = 3.40, *p* < 0.001). Among the control variables, gender significantly and negatively influenced life satisfaction (β = −0.12, *t* = −3.36, *p* < 0.001), and age had no significant effect on goal conflict (β = 0.06, *t* = 1.59, *p* > 0.05).

The bootstrap method was further used to test the mediation effect and estimate the confidence interval, and the results were shown in Table 2. The direct effect of hedonic motivation on life satisfaction was marginally significant [β = 0.108, 95% CI = (−0.004; 0.220), *p* = 0.056], and the total effect [β = 0.065, 95% CI = (−0.045; 0.175)] was not significant. The total indirect effect was significant [β = −0.044, 95% CI = (−0.077; −0.018)], but showed a negative effect in contrast to the nature of the direct effect. Among the three mediating paths of hedonic motivation, the path mediated by goal conflict was not significant [β = −0.008, 95% CI = (−0.034; 0.015)], but the path mediated by mixed emotions [β = −0.018, 95% CI = (−0.042; −0.004)] and the path chain mediated by goal conflict and mixed emotions [β = −0.018, 95% CI = (−0.034; −0.007)] were significant. The direct and indirect paths of hedonic motivation have opposite effect values, and there were suppressing effects between them.

**TABLE 2:** Mediating effect test.

Paths	Effect size	Boot	95% CI	
		**SE**	**LLCI**	**ULCI**
Hedonic motivation→ Life satisfaction (direct effect)	0.108	0.057	-0.004	0.220
Hedonic motivation→ Goal conflict→ Life satisfaction	-0.008	0.012	-0.034	0.015
Hedonic motivation→ Mixed emotions→ Life satisfaction	-0.018*	0.010	-0.042	-0.004
Hedonic motivation→ Goal conflict→ Mixed emotions→ Life satisfaction	-0.018**	0.007	-0.034	-0.007
Total indirect effect of hedonic motivation	-0.044**	0.015	-0.077	-0.018
Total effect of hedonic motivation	0.065	0.056	-0.045	0.175
Eudaimonic motivation →Life satisfaction (direct effect)	0.380***	0.054	0.273	0.485
Eudaimonic motivation→ Goal conflict→ Life satisfaction	0.006	0.010	-0.011	0.028
Eudaimonic motivation→ Mixed emotions→ Life satisfaction	0.025**	0.011	0.009	0.052
Eudaimonic motivation→ Goal conflict→ Mixed emotions→ Life satisfaction	0.013**	0.006	0.004	0.028
Total indirect effect of eudaimonic motivation	0.044***	0.015	0.020	0.079
Total effect of eudaimonic motivation	0.424***	0.052	0.323	0.527

*p < 0.05, **p < 0.01, ***p < 0.001.

The direct effect [β = 0.380, 95% CI = (0.273; 0.485)], total indirect effect [β = 0.044, 95% CI = (0.020; 0.079)], and total effect [β = 0.424, 95% CI = (0.323; 0.527)] of eudaimonic motivation on life satisfaction were significant, and all showed positive effect. Among the three mediating paths of eudaimonic motivation, the path mediated by goal conflict was not significant [β = 0.006, 95% CI = (−0.011; 0.028)], but the path mediated by mixed emotion [β = 0.025, 95% CI = (0.009; 0.052)] and the path chain mediated by goal conflict and mixed emotions [β = 0.013, 95% CI = (0.004; 0.028)] were significant.

The study also compared the effects of the corresponding paths of hedonic and eudaimonic motivation using the bias-correction percentile bootstrap method, and the results were shown in Table 3. There were significant differences between hedonism and eudaimonic motivation in direct effect [β*_*H*–*E*_* = −0.272, 95% CI = (−0.475; −0.073)], indirect effect of mixed emotions [β*_*H–E*_* = −0.044, 95% CI = (−0.091; −0.013)], chain mediating effect of goal conflict and mixed emotions [β*_*H–E*_* = −0.031, 95% CI = (−0.061; −0.012)], total indirect effect [β*_*H–E*_* = −0.088, 95% CI = (−0.152; −0.039)], and total effect [β*_*H–E*_* = −0.360, 95% CI = (−0.555; −0.170)]. There was no significant difference in the indirect effect of goal conflict [β*_*H–E*_* = −0.013, 95% CI = (−0.061; 0.028)].

**TABLE 3:** Comparison of the effects of hedonic motivation and eudaimonic motivation on life satisfaction.

Items for comparison	Difference of effect size (H-E)	Boot SE	95% CI	
			**LLCI**	**ULCI**
Direct effect	−0.272**	0.102	−0.475	−0.073
Indirect effect of goal conflict	−0.013	0.022	−0.061	0.028
Indirect effect of mixed emotions	−0.044**	0.019	−0.091	−0.013
Chain indirect effect of goal conflict and mixed emotions	−0.031***	0.012	−0.061	−0.012
Total indirect effect	−0.088***	0.028	−0.152	−0.039
Total effect	−0.360***	0.099	−0.555	−0.170

*p < 0.05, **p < 0.01, ***p < 0.001.

H-E stood for hedonic motivation minus eudaimonic motivation.

## 4. Discussion

### 4.1. The influence of hedonic and eudaimonic motivation on life satisfaction

In the path analysis of the mediating model, hedonic motivation significantly, and positively influenced life satisfaction (Figure 2). Still, this direct path was found to be only marginally significant when tested for mediation effects using the Bootstrap method (Table 2). Moreover, the total effect of hedonic motivation on life satisfaction was insignificant. In contrast, the direct effect and total effects of eudaimonic motivation were highly significant, both showing a positive effect. It is evident that hedonic motivation is not a robust predictor of life satisfaction compared to eudaimonic motivation, and hypothesis 1 is supported. This result is consistent with previous findings (Ortner et al., 2018; Lin and Chan, 2020; Sun et al., 2022), as well as our theoretical inference based on Self-Determinism Theory (Ryan and Deci, 2017). Hedonic and eudaimonic motivations promote different values and principles. These principles determine which life goals individuals choose to achieve, whether these goals are consistent with social and individual development, whether there is a greater chance of encountering irreconcilable contradictions and obstacles, etc. (Huta and Waterman, 2014; Giuntoli et al., 2021). The answers to these questions influence individual life satisfaction (Ryan and Deci, 2017).

The present study also found that the positive effect of hedonic motivation on life satisfaction was much smaller than that of eudaimonic motivation. From the statistical analysis perspective, this may be closely related to the suppressing effect. By comparing the total indirect effects of the two motivations, it was not difficult to find that the total indirect effect of hedonic motivation, although significant, was negative and had the opposite sign of the direct effect. In contrast, the total indirect effect of eudaimonic motivation was significant and consistent with the nature of the direct effect. The larger suppressing effects between different paths of hedonic motivation may explain the insignificant and much smaller total effects than eudaimonic motivation (MacKinnon et al., 2000). Suppressing effects complicates the effect of hedonic motivation on life satisfaction. At this time, we should not only pay attention to their direct and total effects but also investigate the performance and effects of different mediating paths in detail.

### 4.2. The role of goal conflict

This study found that the path mediated by goal conflict did not significantly influence life satisfaction, and hypothesis 2 was not supported. In the first half of the mediation path, hedonic motivation positively influenced goal conflict, while eudaimonic motivation negatively influenced goal conflict. These effects are consistent with this study’s theoretical speculation based on Self-Determination Theory (Ryan and Deci, 2017; Lin and Chan, 2020). Hedonic motivation is more likely to cause goal conflict than eudaimonic motivation, making it difficult to fully satisfy hedonic needs when hedonic goals are frustrated in the realization process. However, the negative effect of goal conflict on life satisfaction did not reach a significant level, a result that was not consistent with the theoretical expectations of this study (Boudreaux and Ozer, 2013; Corr and Krupić, 2017; Gray et al., 2017). A further review of literature on the relationship between goal conflict and life satisfaction revealed that while most studies found that goal conflict significantly reduced life satisfaction, a subset of studies found that this effect was insignificant (Romero et al., 2009; Segerstrom et al., 2016). Theoretical and empirical studies on multi-goal pursuits have argued that the relationship between goal conflict and positive psychological outcomes is complex and that individual factors and sample characteristics, among others, may affect the relationship between the two (Gray et al., 2017; Carrera et al., 2020). Therefore, there may be some moderating factors between goal conflict and life satisfaction, resulting in different results under different situations or conditions. The negative effect of goal conflict on life satisfaction may require certain mediating factors to work (Sun et al., 2021). At this time, it is essential to investigate the mediating effect of mixed emotions and the chain mediating effect of goal conflict and mixed emotions.

### 4.3. The mediating role of mixed emotions

The test for mediating effect showed that the effect of mixed emotions as a mediator was significant for both motivations. The hedonic motivation influenced life satisfaction significantly and was negatively mediated by mixed emotions, while the opposite was true for eudaimonic motivation, and hypothesis 3 was verified. The relationship between the variables involved in this path is consistent with previous theoretical and empirical research findings (Bee and Madrigal, 2013; Mejía and Hooker, 2017; Sun et al., 2021). According to the Levels of Valence Model and the Co-activation Model of Healthy Coping, it can be inferred that, unlike eudaimonic motivation, hedonic motivation promotes a happiness pursuit process that does contain both positive and negative valence components and the resulting high mixed emotional experience has a negative impact on the individual’s life satisfaction (Larsen et al., 2003; Shuman et al., 2013). Although timely enjoyment can be pleasurable, it is transient, and individuals need to constantly seek out positive events to maintain pleasant feelings. This process is not always successful, as positive events that bring relaxation and satisfaction are not readily available. So, hedonic motivation often induces simultaneous experiences of positive and negative emotions (mixed emotions). The co-occurrence of opposite valence emotions can lead to varying degrees of ambivalence and discomfort, leading to negative decisions and adverse experiences. It is an essential antecedent to individuals’ negative evaluations of their life.

### 4.4. The chain mediating effect of goal conflict and mixed emotions

The more important finding of this study was that hedonic motivation significantly and negatively influenced life satisfaction through the chain mediation of goal conflict and mixed emotions. In contrast, eudaimonic motivation positively influenced life satisfaction through that. The results supported hypothesis 4. This result is consistent with the findings of previous studies on related variables (Bee and Madrigal, 2013; Mejía and Hooker, 2017; Sun et al., 2021), as well as with the Self-Determination Theory, the Levels of Valence Model, and the Co-activation Model of Health Coping (Larsen et al., 2003; Shuman et al., 2013; Ryan and Deci, 2017). The pleasure and comfort goals sought by hedonistic motivation are less aligned with individual development and social expectations than eudaimonic motivation. Highly hedonic individuals need to accomplish academic, work, and organizational goals while pursuing hedonic goals. The ambivalence of the two types of goals in terms of motivation and ultimate purpose causes individuals to experience more goal conflicts (Ryan and Deci, 2017; Kryza-Lacombe et al., 2019). These goal conflict situations include positive valence (e.g., benefits from achieving goals) and negative valence (e.g., goals interfering with each other). Moreover, both valences are essential to individual behavior and have similar weight in the evaluation system of emotions, so they can enter into the processing of emotions simultaneously and contribute to a higher mixed emotional experience of individuals (Shuman et al., 2013). Higher mixed emotional experience in goal-seeking contexts tends to be detrimental to fast decision-making, efficient goal behavior, and goal achievement. When individuals’ hedonic goals are not achieved, and hedonic needs are not met, their life satisfaction decreases (Larsen et al., 2003; Mejía and Hooker, 2017; Ryan and Deci, 2017).

### 4.5. Reasons why hedonists are less happy than eudaimonists

According to Self-Determination Theory, the direct reason why hedonists are less happy than eudaimonists may be related to the fact that their hedonic needs are not fully met (Ryan and Deci, 2017). The mechanism behind this immediate cause may involve three levels of conflict and the resulting emotional experience. First, the goals pursued by hedonic motivation and individual development requirements are contradictory at the individual level. Any personal development in cognition and abilities requires effort and may even involve overcoming difficulties and obstacles from within and outside the individual. This requirement is consistent with the notion of eudaimonic motivation to achieve happiness through struggle. Still, it conflicts with hedonistic motivation’s single-minded pursuit of pleasure and avoidance of pain (Kryza-Lacombe et al., 2019). Second, at the organizational level, the alignment of individual and organizational goals is essential for the rapid integration of individuals into the organization and their career development (Ardıç et al., 2016). A purely hedonistic goal is often inconsistent with the organization’s requirement to work hard and serve the interests of the organization. Third, at the social level, the public does not encourage a life that aims purely at pleasure, comfort, and enjoyment (Yang et al., 2017; Gentzler et al., 2021). This tendency is more prominent in the eastern cultural context, which emphasizes collectivism. For example, in China, people tend to admire strivers who aim for self-actualization and the pursuit of meaning and despise hedonists who seek for mere comfort and enjoyment (Zhao et al., 2015). The hedonic motivation is inconsistent with the requirements of the three levels, so hedonists encounter more goal conflicts in pursuing happiness than eudaimonists. According to the results of the present study, while these conflicts will not directly lead to a decrease in life satisfaction, they induce more intense mixed emotional experiences that indirectly impair life satisfaction.

### 4.6. Contributions and limitations

In terms of the relationship between hedonic and eudaimonic motivation and life satisfaction, previous studies have mainly used the degree of correlation to determine the magnitude of the role of different motivations (Huta and Ryan, 2010; Ortner et al., 2018; Gentzler et al., 2021). This approach can uncover the complexity of the relationship between hedonic motivation and life satisfaction but fails to examine the causes of this complex relationship (Sun et al., 2022). From the perspective of goal pursuit and using goal conflict and mixed emotions as mediating variables, this study attempts to explain why hedonists are less happy than eudaimonists by comparing the differences between the two happiness motivations in terms of direct, indirect, and total effects. The ideas and results of the study help inspire scholars to conduct more in-depth and detailed research on the internal mechanism of individuals’ pursuit of happiness. In addition, the deficiencies of hedonic motivation and the advantages of eudaimonic motivation presented by the study provide directions for the cultivation of happiness motivation for adolescents in the practice field. The study’s results suggest that it is inappropriate for educators to simply encourage adolescents to seek happiness through relaxation, comfort, and avoidance of pain. Instead, educators should focus on teaching adolescents how to balance recreation and study and turn eudaimonic motivational goals such as personal development and social needs into the primary source of their happiness.

The potential limitations of this study may involve three aspects. First, the process from motivation to individual behavior to happy outcomes is complex. The present study, based on a tentative exploration from the perspective of goal pursuit, focused on the role of the relationship between goals and the corresponding emotional experience in it. In contrast, factors such as goal attainment and need satisfaction, closely related to goal pursuit and life satisfaction, were not included in the existing model. Future research should consider including more critical variables based on theories related to motivation and goals, constructing a more comprehensive model of influence mechanisms, and developing and improving the existing research findings. Second, this study focused on the differences in effects and mechanisms between hedonic and eudaimonic motivation in Chinese groups and did not include cultural differences in the model. Western cultures are more accepting of hedonism than Eastern cultures (Ortner et al., 2018; Lin and Chan, 2020). So the goal conflict and mixed emotions associated with hedonic motivation may be relatively weak, and the resulting differences in effects between hedonic motivation and eudaimonic motivation may not be significant. Future research can examine the generalizability of the model constructed in this study by comparing Eastern and Western groups. Finally, the study did not find a mediating role for goal conflict. Based on the analysis, other moderating variables besides the mediating role of mixed emotions may be involved between goal conflict and life satisfaction, such as the individual’s construal level (Carrera et al., 2020), ability and strategy to cope with conflict (Kung and Scholer, 2020). Therefore, adding these essential individual difference variables to examine changes on this path could be considered in the future.

## 5. Conclusion

In contrast to eudaimonic motivation, hedonic motivation does not robustly influence individual life satisfaction. From the perspective of goal pursuit, the reasons for this may involve two crucial variables: goal conflict and mixed emotion. The chain mediation model constructed for this study found that (1) the direct effect of hedonic motivation on life satisfaction was marginally significant, and the effect size was much smaller than that of eudaimonic motivation. (2) The direct and indirect effects of hedonic motivation are opposite, with a large suppressing effect. In contrast, all paths of eudaimonic motivation positively affected life satisfaction. (3) Hedonic motivation negatively influenced life satisfaction through mixed emotions and the chain mediating effect of goal conflict and mixed emotions, whereas eudaimonic motivation positively influenced life satisfaction through these two mediating paths. (4) The effects on all paths of hedonic motivation were significantly smaller than those of eudaimonic motivation, except for the path mediated by goal conflict. Thus, the fact that hedonists are less happy than eudaimonists is indeed related to the fact that they experience more goal conflict; as a result, they have more mixed emotional experiences.

## Data availability statement

The datasets presented in this study can be found in online repositories. The names of the repository/repositories and accession number(s) can be found in the article/Supplementary material.

## Ethics statement

The studies involving human participants were reviewed and approved by Human Subjects Ethics Branch of the Beijing Union University Research Committee. The patients/participants provided their written informed consent to participate in this study.

## Author contributions

GW contributed to the conception and design of the study. LL and WS performed the statistical analysis. WS wrote the first draft of the manuscript. PF and YJ revised it critically for important intellectual content. GW and XD collected the raw data and organized the database. All authors contributed to the article and approved the submitted version.

## Footnotes

1
https://www.wjx.cn/

## References

[B1] Ardıç K. Uslu O. Oymak Ö Özsoy E. Özsoy T. (2016). Comparing person organization fit and person job fit. *J. Econ. Manag. Sci.* 25 5–13. 10.22367/jem.2016.25.01

[B2] Bee C. C. Madrigal R. (2013). Consumer uncertainty: The influence of anticipatory emotions on ambivalence, attitudes, and intentions. *J. Consum. Behav.* 12 370–381. 10.1002/cb.1435

[B3] Berrios R. Totterdell P. Kellet S. (2015a). Investigating goal conflict as a source of mixed emotions. *Cogn. Emot.* 29 755–763. 10.1080/02699931.2014.939948 25040183

[B4] Berrios R. Totterdell P. Kellett S. (2015b). Eliciting mixed emotions: A meta-analysis comparing models, types, and measures. *Front. Psychol.* 6:428. 10.3389/fpsyg.2015.00428 25926805 PMC4397957

[B5] Berrios R. Totterdell P. Kellett S. (2018). When feeling mixed can be meaningful: The relation between mixed emotions and eudaimonic well-being. *J. Happiness Stud.* 19 841–861. 10.1007/s10902-017-9849-y

[B6] Boudreaux M. J. Ozer D. J. (2013). Goal conflict, goal striving, and psychological wellbeing. *Motiv. Emot.* 37 433–443. 10.1007/s11031-012-9333-2

[B7] Braaten A. Huta V. Tyrany L. Thompson A. (2019). Hedonic and eudaimonic motives toward university studies: How they relate to each other and to well-being derived from school. *J. Posit. Psychol. Wellbeing* 3 179–196. 10.19090/pp.2014.1.5-21

[B8] Carrera P. Fernández I. Muñoz D. Caballero A. (2020). Using abstractness to confront challenges: How the abstract construal level increases people’s willingness to perform desirable but demanding actions. *J. Exp. Psychol.* 26 339–349. 10.1037/xap0000244 31535885

[B9] Corr P. J. Krupić D. (2017). Chapter two -motivating personality: Approach, avoidance, and their conflict. *Adv. Motiv. Sci.* 4 39–90. 10.1016/bs.adms.2017.02.003

[B10] Diener E. D. Emmons R. A. Larsen R. J. Griffin S. (1985). The satisfaction with life scale. *J. Pers. Assess.* 49 71–75. 10.1207/s15327752jpa4901_13 16367493

[B11] Emmons R. A. King L. A. (1988). Conflict among personal strivings: Immediate and long-term implications for psychological and physical well-being. *J. Pers. Soc. Psychol.* 54 1040–1048. 10.1037/0022-3514.54.6.1040 3397863

[B12] Gentzler A. L. Delong K. L. Palmer C. A. Huta V. (2021). Hedonic and eudaimonic motives to pursue well-being in three samples of youth. *Motiv. Emot.* 45 312–326. 10.1007/s11031-021-09882-6

[B13] Giuntoli L. Condini F. Ceccarini F. Huta V. Vidotto G. (2021). The different roles of hedonic and eudaimonic motives for activities in predicting functioning and well-being experiences. *J. Happiness Stud.* 22 1657–1671. 10.1007/s10902-020-00290-0

[B14] Gray J. S. Ozer D. J. Rosenthal R. (2017). Goal conflict and psychological well-being: A meta-analysis. *J. Res. Pers.* 66 27–37. 10.1016/j.jrp.2016.12.003

[B15] Huta V. Ryan R. M. (2010). Pursuing pleasure or virtue: The differential and overlapping well-being benefits of hedonic and eudaimonic motives. *J. Happiness Stud.* 11 735–762. 10.1007/s10902-009-9171-4

[B16] Huta V. Waterman A. S. (2014). Eudaimonia and its distinction from hedonia: Developing a classification and terminology for understanding conceptual and operational definitions. *J. Happiness Stud.* 15 1425–1456. 10.1007/s10902-013-9485-0

[B17] Kryza-Lacombe M. Tanzini E. O’Neill S. (2019). Hedonic and eudaimonic motives: Associations with academic achievement and negative emotional states among urban college students. *J. Happiness Stud.* 20 1323–1341. 10.1007/s10902-018-9994-y 31656399 PMC6813844

[B18] Kung F. Y. H. Scholer A. A. (2020). The pursuit of multiple goals. *Soc. Pers. Psychol. Compass* 14:e12509. 10.1111/spc3.12509

[B19] Larsen J. T. (2017). Introduction to the special section on mixed emotions. *Emot. Rev.* 9 97–98. 10.1177/1754073916672523

[B20] Larsen J. T. Hemenover S. H. Norris C. J. Cacioppo J. T. (2003). “Turning adversity to advantage: On the virtues of the coactivation of positive and negative emotions,” in *A psychology of Human Strengths: Fundamental Questions and Future Directions for a Positive Psychology*, eds Aspinwall L. G. Staudinger U. M. (Washington, DC: American Psychological Association Press), 211–225. 10.1037/10566-015

[B21] Lin L. Chan H. (2020). The associations between happiness motives and well-being in China: The mediating role of psychological need satisfaction and frustration. *Front. Psychol.* 11:2198. 10.3389/fpsyg.2020.02198 33013570 PMC7495498

[B22] MacKinnon D. P. Krull J. L. Lockwood C. M. (2000). Equivalence of the mediation, confounding, and suppression effect. *Prev. Sci.* 1 173–181. 10.1023/A:1026595011371 11523746 PMC2819361

[B23] Mejía S. T. Hooker K. (2017). Mixed emotions within the context of goal pursuit. *Curr. Opin. Behav. Sci.* 15 46–50. 10.1016/j.cobeha.2017.05.015 29201977 PMC5703421

[B24] Ortner C. Corno D. Fung T. Y. Rapinda K. (2018). The roles of hedonic and eudaimonic motives in emotion regulation. *Pers. Individ. Differ.* 120 209–212. 10.1016/j.paid.2017.09.006

[B25] Podsakoff P. M. MacKenzie S. B. Lee J. Y. Podsakoff N. P. (2003). Common method biases in behavioral research: A critical review of the literature and recommended remedies. *J. Appl. Psychol.* 88 879–903. 14516251 10.1037/0021-9010.88.5.879

[B26] Romero E. Villar P. Luengo M. Á Gómez-Fraguela J. A. (2009). Traits, personal strivings and well-being. *J. Res. Pers.* 43 535–546. 10.1016/j.jrp.2009.03.006

[B27] Ryan R. M. Deci E. L. (2017). *Self-Determination Theory: Basic Psychological Needs in Motivation, Development, and Wellness.* New York, NY: Guilford Press.

[B28] Segerstrom S. C. Jones A. C. Scott A. B. Crofford L. J. (2016). Daily goals and psychological well-being in midlife and older women: Physical pain interacts with goal conflict. *Res. Hum. Dev.* 13 328–341. 10.1080/15427609.2016.1234306 28603467 PMC5464603

[B29] Sheldon K. M. Lyubomirsky S. (2012). The challenge of staying happier: Testing the hedonic adaptation prevention model. *Pers. Soc. Psychol. Bull.* 38 670–680. 10.1177/0146167212436400 22361725

[B30] Shuman V. Sander D. Scherer K. R. (2013). Levels of valence. *Front. Psychol.* 4:261. 10.3389/fpsyg.2013.00261 23717292 PMC3651968

[B31] Sun W. Liu L. Zheng Z. Jiang Y. Fang P. (2022). Why eudemonia bring more happiness: The multiple mediating roles of meaning of life and emotions. *Curr. Psychol.* 10.1007/s12144-022-03058-2

[B32] Sun W. Zheng Z. Jiang Y. Tian L. Fang P. (2021). Does goal conflict necessarily undermine wellbeing? a moderated mediating effect of mixed emotion and construal level. *Front. Psychol.* 12:653512. 10.3389/fpsyg.2021.653512 34149538 PMC8206492

[B33] Tandler N. Krauss A. Proyer R. T. (2020). Authentic happiness at work: Self- and peer-rated orientations to happiness, work satisfaction, and stress coping. *Front. Psychol.* 11:1931. 10.3389/fpsyg.2020.01931 32849134 PMC7426460

[B34] Waterman A. S. (2007). On the importance of distinguishing hedonia and eudaimonia when contemplating the hedonic treadmill. *Am. Psychol.* 62 612–613. 10.1037/0003-066X62.6.612 17874913

[B35] Yang Y. Li P. Yu K. (2017). Orientations to happiness and subjective well-being in Chinese adolescents. *J. Happiness Stud.* 10 1–17. 10.1007/s12187-016-9410-2

[B36] Zhao H. Xu Y. Wang F. Jiang J. Zhang X. Wang X. (2015). Chinese adolescents’ coping tactics in a parent-adolescent conflict and their relationships with life satisfaction: The differences between coping with mother and father. *Front. Psychol.* 6:1572. 10.3389/fpsyg.2015.01572 26528224 PMC4606045

